# Hemodynamic and Hydrodynamic Pathophysiology in Chiari Type 1 Malformations: Towards Understanding the Genesis of Syrinx

**DOI:** 10.3390/jcm12185954

**Published:** 2023-09-13

**Authors:** Cyrille Capel, Romaric Lantonkpode, Serge Metanbou, Johann Peltier, Olivier Balédent

**Affiliations:** 1 Department of Neurosurgery, Hospital University Center of Amiens-Picardie, 80054 Amiens, Francepeltier.johann@chu-amiens.fr (J.P.); 2 Chimère UR 7516, Jules Verne University, 80000 Amiens, France; olivier.baledent@chu-amiens.fr; 3 Radiology Department, Hospital University Center of Amiens-Picardie, 80054 Amiens, France; 4 Image Processing Department, Hospital University Center of Amiens-Picardie, 80054 Amiens, France

**Keywords:** Chiari malformation type 1, syringomyelia, cerebrospinal fluid, hydrodynamic, cine-MRI

## Abstract

Background: The pathophysiology of this association of type 1 Chiari malformation (CM1) and syrinxes is still unknown. There is an alteration in the dynamics of neurofluids (cerebrospinal fluid, arterial and venous blood) during the cardiac cycle in CM1. Our objective is to quantify CSF or arterial blood or venous blood flow in patients with Chiari syndrome (CS) with and without syrinxes using phase-contrast MRI (PCMRI). Methods: We included 28 patients with CM1 (9 with syrinxes, 19 without). Morphological MRI with complementary PCMRI sequences was performed. We analyzed intraventricular CSF, subarachnoid spaces CSF, blood, and tonsillar pulsatility. Results: There is a highly significant correlation (*p* < 0.001) between cerebral blood flow, cerebral vascular expansion volume and venous drainage distribution. Venous drainage distribution is significantly inversely correlated with oscillatory CSF volume at the level of the foramen magnum plane [−0.37 (0.04)] and not significantly correlated at the C2C3 level [−0.37 (0.05)] over our entire population. This correlation maintained the same trend in patients with syrinxes [−0.80 (<0.01)] and disappeared in patients without a syrinx [−0.05 (0.81)]. Conclusion: The distribution of venous drainage is an important factor in intracranial homeostasis. Impaired venous drainage would lead to greater involvement of the CSF in compensating for arterial blood influx, thus contributing to syrinx genesis.

## 1. Introduction

Chiari type I malformations have a radiological definition based on ptosis of the cerebellar tonsils beyond the foramen magnum [1,2,3,4]. Clinical presentations are multiple and many symptoms are nonspecific [3,5]. Frìc proposes to reserve the term Chiari malformation for types II, III and IV and to call type 1 Chiari as “Chiari syndrome” [6]. In this study, we will use the term Chiari syndrome (CS). The malformative character is strongly questioned. The pathophysiological mechanisms are not well understood. Many studies suggest alterations in craniospinal hydrodynamics in CS [4,7,8].

Under physiological conditions and during each cardiac cycle, the interactions between cerebrospinal fluid (CSF) and arterial and venous blood are well known [9,10,11,12,13]. There is a sudden entry of arterial blood volume that is not instantly compensated by cerebral venous drainage. Thus, there is a transient increase in intracranial vascular volume at systolic time of the cardiac cycle. This transient volume increase less than 1 mL [12] is not completely [10,12] and instantly compensated by the flushing of CSF from the intracranial subarachnoid (SAS) toward the spinal SAS passing by the unique and narrow pathway of the foramen magnum. This flushing of CSF through the foramen magnum with each cardiac cycle can be considered as a mobile compliance [12] of the intracranial compartment’s neurofluid expansion.

Intracranial venous drainage increases in the diastolic phase to compensate for the systolic arterial blood brain volume expansion. Thus, intracranial pressure decreases and leads to reversed CSF flow from the spinal to the intra cranial compartment. These rapid and physiological back and forth movements of the CSF are continuously active during each cardiac cycle, even if instantaneously intracranial neurofluid volume is not constant. It is important to note that over the mean time of the cardiac cycle the intracranial volume remains unchanged. The time scale of cardiac cycles is shorter than the time scale of the Monro–Kellie doctrine, which describes preservation of mean intracranial volume [12,13]. This doctrine states that intracranial volume components are uncompressible and intracranial volume is constant. Thus, any addition of volume in one of the intracranial compartments must be balanced by removing the same volume from the other compartments. We hypothesize that, during cardiac cycles, this doctrine is not true. We know that CSF oscillations do not completely balance arteriovenous intracranial pulsations and lead to the existence of small but not insignificant intracranial volume change (IVC). IVC is the origin of ICP amplitude changes during the cardiac cycle. We hypothesize that if CSF or cerebral venous flows are not well synchronized with arterial flow during cardiac cycles, then oscillating intracranial pressure amplitude can increase and maintain the same mean ICP. That could initiate oscillated compensating movements on the cerebellar tonsils through the foramen magnum and create a vicious circle by increasing the foramen magnum’s CSF flow resistance.

Phase-contrast MRI (PCRMI) is a unique non-invasive tool to quantify CSF and venous and arterial blood flow dynamics in the craniospinal system during one cardiac cycle [9,14,15]. By using multiple slice planes, positioned perpendicular to the flow axis of the compartment under study, and by adapting encoding velocity and retrospective cardiac gating, it is possible to measure velocity variation voxel by voxel over the course of a cardiac cycle. The semi-automatic segmentation we use allows us to determine the surface area of these compartments and thus measure flow variations. This procedure enables us to measure the flow variations of the compartments and to understand the interactions of these compartments with each other [12].

In the context of CS, cervical CSF flushing may be impaired. This CSF compensatory mechanism in response to intracranial systolic vascular volume expansion is therefore altered, leading to probable alteration of intracranial pressure [8]. In CS, there is a restriction of the CSF outflow area at the SAS [2,16]. This decrease in flow area increases the resistance to flow at this level [16]. This induces acceleration of flow velocities [17,18,19]. Nevertheless, according to Poiseuille’s law, if the resistance increases, the pressure difference must also increase to generate the same flow. If this adaptation is not possible, to avoid the pressure increasing, the system can induce a pulsatility of the neuraxis at the level of the foramen magnum synchronized with the cardiac cycle to add complementary compliance to the CSF. This would be the main marker of symptomatic CS [20]. This pulsatility is well described in each region of the foramen magnum [19]. It has been proposed in many studies that in CS there is a pressure gradient between the intracranial and cervical SAS secondary to CSF oscillations at the foramen magnum disturbance [7,8,16,21]. The increase in this pressure gradient would lead to a change in the morphology of the cerebellum and the development of biomechanical forces altering the constitution of the tissues and the vascularization [15]. This leads to abnormal tissue pulsatility and changes in local vascularization and is thought to be the cause of syrinx formation, often through repermeabilization of the obex (lower orifice of the IVth ventricle). It can also lead to anatomical changes, such as an increase in the volume of the IVth ventricle [22,23]. The analysis of hemohydrodynamics therefore appears to be perfectly complementary to morphological radiological analysis. More recently, a modification of cerebral hydrodynamics in CS has been described, as well as hemodynamic alterations, in particular alterations in venous drainage [24].

The association of CS with a syrinx has been the subject of many pathophysiological proposals [25]. The first proposal was based on the repermeabilization of the obex. This phenomenon would be secondary to a structural modification at the level of the obex through the pulsatility of CSF [26]. In the 1980s, it was proposed that the syrinx was secondary to a pressure gradient of CSF between the intracranial and cervical SAS on either side of the foramen magnum [27]. In the 1990s, Oldfield introduced an important dynamic concept [7]. The restriction of the SAS at the level of the foramen magnum generates a piston effect of the tonsils. This would be secondary to the disturbance of the pulsatility of the CSF at the level of the foramen magnum. The tonsils would play a role in transmitting this pulsatility, which could participate in the genesis of the syrinx.

These mechanisms are present in many cases of CS with an associated or unassociated syrinx. Associated factors must exist. A few studies have highlighted the possibility of a hemodynamic origin to syrinxes. These hemodynamic disorders would potentially be related to venous drainage [24,28].

Our objective is to show and quantify by PCMRI the existence of CSF or arterial blood or venous blood flow in patients with CS with and without syrinxes.

## 2. Patients and Methods

### 2.1. Patients

This is a retrospective observational study in two populations of patients with Chiari malformation with and without syrinxes.

Twenty-eight patients older than 18 years of age (37 ± 13 (19–74) years) with CS were retrospectively included from 2017 to 2022. The symptomatology was secondary to CS and confirmed after examination by a neurosurgeon. Patients with associated spina bifida (Chiari malformations type II) and those younger than 18 years of age were excluded.

The diagnostic workup required brain and spinal cord MRI, which allowed the patients to be divided into two groups: the syrinx group and the non-syrinx group.

A total of 9 patients had an associated syrinx (CS/syrinx group) and 19 patients were included in the group of patients without a syrinx (CS group).

### 2.2. PCMRI Acquisition

All patients underwent a 3T MRI investigation of the head and spine based on the morphological sequences of the conventional diagnostic workup. Complementary phase-contrast sequences were added to investigate CSF and blood flows.

**Figure 1:**
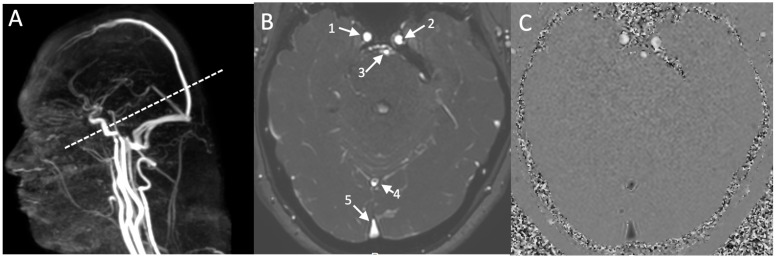
(**A**) Cross-sectional plane of vascular acquisitions by phase-contrast sequence MRI (PCMRI). PCMRI produced 32 amplitude images and 32 phase images in which pixel intensities are functions of the velocities of the blood. (**B**) One of the amplitudes images of the cardiac cycle: 1—right carotid artery; 2—basilar artery; 3—left carotid artery; 4—straight sinus; 5—superior sagittal sinus. (**C**) Phase image showing the arterial flows in white and the sinus flows in black.

**Figure 2:**
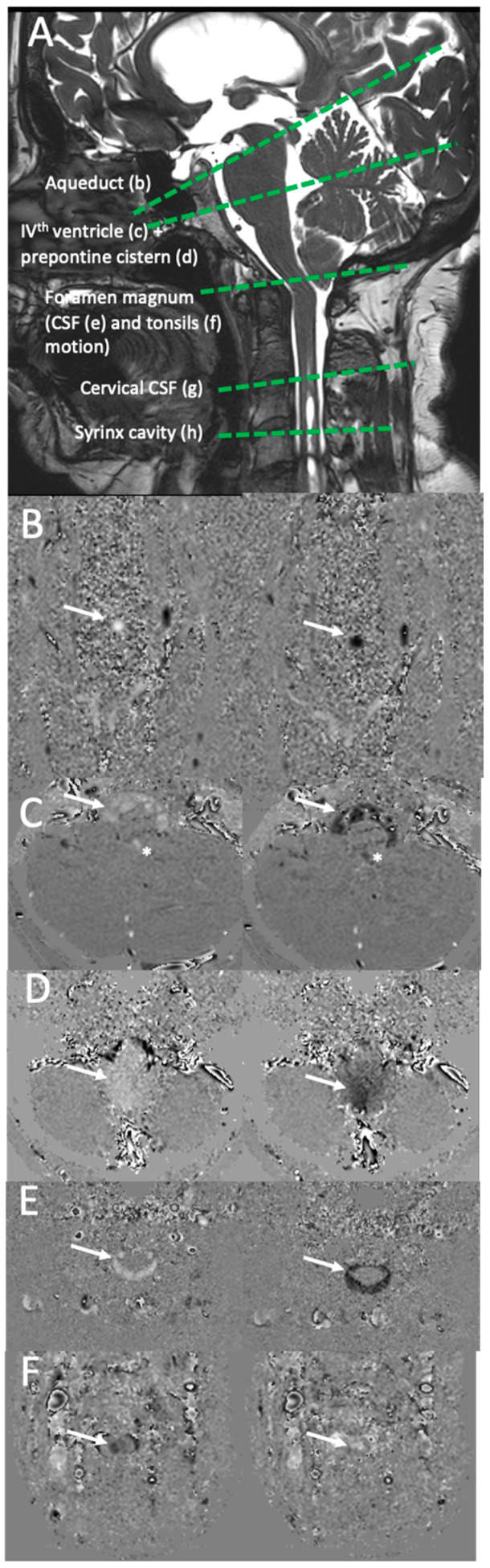
Plane position and phase-contrast images. (**A**) Cross-sectional plane of phase-contrast MRI acquisitions for the evaluation of craniospinal hydrodynamics. The cross-sectional planes were placed on a BFFE sagittal slice. Aqueduct: slice plane for analysis of cerebrospinal fluid (CSF) dynamics within the aqueduct of Sylvius; PPC (prepontine cistern): slice plane for analysis of subarachnoid CSF dynamics of the prepontine cisterns; FM (foramen magnum): slice plane for analysis of the dynamics of the subarachnoid CSF at the foramen magnum and analysis of the dynamics of the cerebellar tonsils; cervical CSF: analysis of the dynamics of the subarachnoid CSF at the level of the C2C3 disc. (**B**) Aqueductal phase-contrast acquisition (velocity encoding (Venc = 10 cm/s). The aqueduct is indicated by the arrow. The white signal represents caudocranial displacement of CSF and the black signal represents craniocaudal displacement. These signals represent 2 of the 32 phases of one cardiac cycle. (**C**) Phase-contrast acquisition at the level of the prepontine cistern (arrow) and IVth ventricle (star) (Venc = 5 cm/s). These signals represent 2 of the 32 phases of one cardiac cycle. (**D**) Phase-contrast acquisition through the foramen magnum plane (Venc = 2 cm/s). The displacement of the tonsils (arrow) is shown by the white signal (caudocranial displacement) and the black signal (craniocaudal displacement). These signals represent 2 of the 32 phases of one cardiac cycle. (**E**) Cervical CSF phase-contrast acquisition (Venc = 5 cm/s). The displacement of upper cervical subarachnoidal CSF is shown by the white signal (caudocranial displacement) and the black signal (craniocaudal displacement). These signals represent 2 of the 32 phases of one cardiac cycle. (**F**) Syrinx phase-contrast acquisition (Venc = 5 cm/s). The displacement of CSF into the syrinx is shown by the white signal (caudocranial displacement) and the black signal (craniocaudal displacement). These signals represent 2 of the 32 phases of one cardiac cycle.

The planes were positioned perpendicular to the axis of the studied flow regions. Vascular investigations were performed at the intracranial level for the internal carotid and the arteries and through the straight and superior sagittal sinuses (Figure 1). CSF investigations were conducted through the aqueduct, the prepontine cistern (PPC), the foramen magnum (FM), the high cervical spinal SAS (at the level of the C2C3 intervertebral disc) and at the level of the syrinx, if present (Figure 2). Cerebellar tonsil movements were evaluated at the level of the foramen magnum.

The PCMRI sequence parameters are presented in Table 1. The encoding speed was selected at 60 cm/s for the blood flows, 10 cm/s for the CSF in the aqueduct, 5 cm/s for the other CSF flows and 2 cm/s for cerebellar tonsil movements. To synchronize PCMRI acquisitions with cardiac cycle, a plethysmograph was added to the finger of the patient.

### 2.3. Data Analysis

Data were analyzed using our in-house software (public), https://www.flow-software.com. Semi-automatic segmentation was used for delineation of the regions of interest: the blood flows, the CSF oscillations and the tissues oscillations. By multiplying the area of the regions of interest by the velocities, flow dynamic curves were calculated over the 32 points of the cardiac cycle.

For the oscillating flows of CSF and the movement of tissues, their stroke volume (SV) was calculated. SV represents the volume displaced through the region of interest during the cardiac cycle [29] both in the cranio-caudal and caudo-cranial direction.

Cerebral arterial blood flow (CBF_a_) was defined as the sum of the flow through the internal carotid and basilar artery. Cerebral venous blood flow (CBF_v_) was measured as the sum of the flow of the straight sinus and superior sagittal sinus. As CBF_v_ does not drain all the CBF itself (other peripherical veins are involved, such as epidurals, etc.), a venous correction factor (α factor) was calculated equal to the mean(CBF_a_)/mean(CBF_v_). It was thus possible to provide a theorical _corrected_CBFv flow curve = α factor × CBF_v_ flow. When this venous factor is equal to 1, _corrected_CBF_v_ is equal to CBF_v_. This corresponds to exclusive jugular venous drainage. When the correction factor is >1, it shows the magnitude of accessory drainage paths.

To evaluate how the cerebral blood volume increases during the cardiac cycle, the cerebral arteriovenous flow curve was calculated by subtraction of CBF_a_ and _corrected_CBF_v_. The arteriovenous stroke volume was calculated as the CSF stroke volume.

The peak amplitude of the flow curves was calculated.

The study parameters were:

- SV_CSF-aqu_, which corresponding to the stroke volume of CSF oscillating through the aqueduct during one cardiac cycle.
- SV_CSF-FM_, which corresponds to the stroke volume of CSF oscillating through the foramen magnum during one cardiac cycle.
- SV_tonsils_, which corresponds to the stroke volume of the tonsils that oscillates through the foramen magnum during one cardiac cycle. This is due to the pulsatility of the tonsils.
- SV_CSF-PPC_, which corresponds to the stroke volume of CSF oscillating through the prepontine cistern (PPC) during one cardiac cycle.
- SV_CSF-cerv_, which corresponds to the stroke volume of CSF oscillating through the upper cervical SAS at the level of the C2C3 intervertebral disc during one cardiac cycle.
- SV_syrinx_, which corresponds to the stroke volume of CSF oscillating into the syrinx during one cardiac cycle.
- CBFa, which represents mean cerebral arterial blood flow during one cardiac cycle.
- SV_blood_, which represents the volume of blood oscillating into the cranial compartment during one cardiac cycle.
- Venous correction factor α, which is the ratio between CBFa and mean venous flow. It corresponds to the cerebral venous drainage repartition. When the α factor is 1, there is exclusive sinus drainage. When the α factor is greater than one, it means that the accessory venous drainage pathway contributes to the sinuses to drain all cerebral blood flow. According to the example, if the α factor equals 2, that means that the sinuses participate in only 1/2 (or 50%) of total cerebral drainage.

### 2.4. Statistic Analysis

We used Xstat for statistical analysis.

Mann–Whitney tests were used to compare hemodynamic parameters between patients with a syrinx and those without. The threshold of significance was set to a 0.05 *p*-value.

The correlation study was performed using a Pearson correlation and linear regression. The threshold of significance was set to a 0.05 *p*-value.

## 3. Results

### 3.1. Patients

All results are presented in Table 2.

### 3.2. Comparison of Hemoydrodynamic Parameters in the Syrinx and Non-Syrinx Groups

There were no differences found between the group of CS patients without a syrinx and the group of patients with syrinxes. All results are shown in Table 3.

### 3.3. Hemodynamic and Hydrodynamic Interactions

Across the overall population, the α factor and the CBF parameters were slightly correlated (R^2^ = 0.16; *p* < 0.001); SV_blood_ and CBFa were slightly correlated (R^2^ = 0.3; *p* < 0.001).

Only one hydrodynamic parameter correlates with SV_blood_, namely SV_CSF-cerv_ in the CS population without a syrinx, where the correlation was high (R^2^ = 0.67; *p* < 0.01). This was less correlated across the overall CS population (R^2^ = 0.50; *p* < 0.01) and no significant correlation was found in the CS with a syrinx population (R^2^ = 0.04; *p* = 0.89). Results are presented in Table 3.

A significant negative correlation was found between the α factor in the CS group with syrinxes (R^2^ = −0.80; *p* < 0.01). This correlation is not present in the CS group without syrinxes (R^2^ = −0.05; *p* = 0.81). All results are shown in Table 4 and Table 5.

## 4. Discussion

Our study provides a quantitative analysis of blood and CSF flows at the craniospinal level through the use of PCMRI in CS patients with and without an associated syrinx. The sequences added to the morphological protocols add about 10 min to the examination. Thus, in a few minutes, we have access to different physiological panels that were previously suspected but not yet understood. In our previous article [24], we observed the impact of surgery in CSF, tonsil and venous pulsatility. This analysis was made by comparing each patient’s pre- and post-operative data. Here, we analyzed differences in CSF, arterial and venous pulsatility in two populations: one with an associated syrinx and the other without.

### 4.1. Comparison of Hydrodynamic Parameters in the Syrinx and Non-Syrinx Groups

#### 4.1.1. Tonsil Pulsatility and Compliance Preservation

In our study, no parameter assessing hydrodynamics is different between our two populations. In the control populations, no pulsatility of the cerebellar tonsils was found [4]. In our population, we found pulsatility for these parenchymal structures passing the plane of the foramen magnum in all patients. This pulsatility cumulated with the pulsatility of the CSF at the level of the foramen magnum is of the same order as the pulsatility of the CSF measured at the cervical level. Cerebellar tonsil pulsatility can trace its origin to when intracranial CSF pulsatility does not sufficiently compensate intracranial blood volume change during the cardiac cycle.

Such intracranial blood volume change is related to the delay between arterial and venous peaks flows [8,15,16,21,24].

To preserve or increase the compliance of the intracranial compartment when mobile CSF compliance is limited, additional pulsatility of the cerebellar tonsils in the spinal canal can be helpful. Adequate intracranial compliance is fundamental to preserving the homeostasis of vascular intracranial flows and the regulation of intracranial pressure.

Post-operatively, a decrease in the pulsatility of the tonsils and restoration of the pulsatility of the CSF at the level of the foramen magnum are observed [24]. This allows us to confirm that the restoration of a CSF flow surface makes it possible to ensure that the mobile compliance linked to the CSF is efficient and additionally allows for the pulsatility of the tonsils to be limited.

Our results, in accordance with Oldfield’s theory [7], confirm the importance of CSF pulsatility in the pathophysiology of CS. This confirms the need for analysis of the oscillatory CSF flow between its different compartments of the craniospinal system. Because cerebral blood flows are the main origin of CSF flows, arterial and venous flows should be investigated with CSF flows.

#### 4.1.2. CSF Pulsatility Compensation

Based on analysis of the CSF velocity profiles, it is largely described that there is essentially an increase in systolic CSF velocity at the foramen magnum [17]. This significant difference between CS patients and a control population is logically explained by the need to preserve the oscillatory CSF volume [21]. To keep the same CSF flow when the flow area at the foramen magnum is restricted, CSF velocity must increase.

### 4.2. Hemodynamic and Hydrodynamic Interactions

#### 4.2.1. Hemodynamic and Hydrodynamics Parameter Interactions

CSF responds passively to intracranial volume changes to preserve intracranial pressure [9,12,30]. This is the case in physiology and could be the origin of the problem in pathologies such as normal-pressure hydrocephalus and idiopathic intracranial hypertension [31,32]. There is a correlation between vascular stroke volume and cervical CSF stroke volume at the cervical level. In our study, there was a correlation between these two parameters for CS patients without a syrinx. This shows that the CSF is an almost instantaneous means of compensation for the influx of intracranial arterial blood, unlike venous drainage, which occurs with a greater delay. For CS patients without a syrinx, CSF volume flushing in spinal areas is proportional to changes in intracranial vascular volume (Table 4). In the population of patients with a syrinx, this correlation was no longer present (Table 4). There is therefore an alteration to the mechanisms of regulation of intracranial pressure through the oscillations of the CSF in the spinal canal. This is consistent with the hypothesis of Oldfield [7,28], who proposes that the genesis of syrinxes is related to altered CSF pulsatility.

#### 4.2.2. Venous Drainage and Regulation of Intracranial Volume Changes

**Figure 3:**
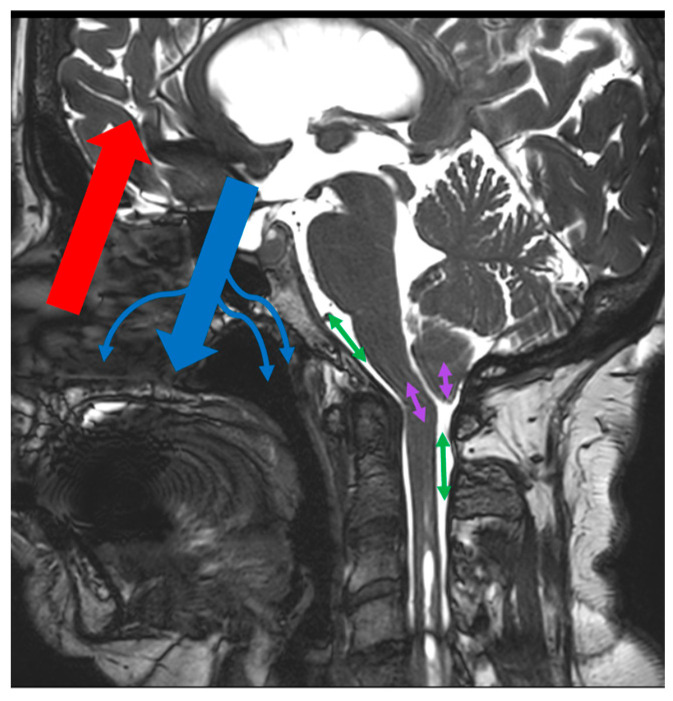
Hemohydrodynamic interactions in Chiari syndrome (CS) with and without a syrinx. During a cardiac cycle, there is a systolic increase in intracranial vascular volume due to a sudden rise in arterial flow (red arrow). There are two compensatory pathways: the first is immediate but incomplete, with cerebrospinal fluid (CSF) flushing into the perimedullary subarachnoid spaces (green arrows); the second is later but complete, with an increase in venous blood flow (blue arrow). CSF flushing is impaired at the foramen magnum in CS. In CS without a syrinx, pulsatility transmission is preserved by CSF pulsatility in the foramen magnum and neuraxis (purple arrows). No hemodynamic changes were observed. In CS with a syrinx, transmission of pulsatility via the pulsatility of the CSF and neuraxis is incomplete. There is an increase in venous drainage (small blue arrows).

The α venous correction factor reflects the participation of alternative drainage routes to the cerebral sinuses as the main source of cerebral venous drainage. In our study, we show that the factor is significantly correlated with cerebral arterial blood flow. As cerebral blood flow increases, so does the involvement of accessory venous drainage pathways. Furthermore, the α venous correction factor presents a negative and significant correlation with the stroke volume of CSF measured at the cervical level in the population of CS patients with a syrinx. No correlation is present in the population of CS patients without a syrinx. The existence of a negative correlation reflects that a decrease in cervical CSF pulsatility (mobile compliance) is related to an increase in the recruitment of accessory drainage pathways in the CS patient population associated with syrinxes. In this population, we also demonstrated that cervical CSF stroke volume was not correlated with vascular stroke volume. There is an alteration to “CSF mobile compliance”. The existence of a negative correlation between the stroke volume of the cervical CSF and the α venous correction factor may be a result of the implementation of new parameters of compensation for the incompetence of the CSF in its compliance role. The increase in the α venous correction factor would allow for the capacities of the system to be increased to compensate for the intracranial vascular volume changes during systole (Figure 3).

#### 4.2.3. Arteriovenous Delay and Venous Drainage Alteration

In 2000, a study by Pinna [28] performed a temporal analysis of CSF flow in the cervical SAS through the use of PCMRI. It was found that the duration of the systolic phase of the CSF dynamics was increased compared to the duration of the diastolic phase in CS patients with an associated syrinx. The CSF is a passive fluid, and its mobilization responds to pressure gradients secondary to arteriovenous exchange. This finding of disturbed temporal distribution is therefore a response to arteriovenous volume variations. Heiss [21] showed through study of intracranial, cervical and lumbar subarachnoid pressure and intraoperative Doppler analysis that there is a peak in cervical subarachnoidal pressure at the earliest phase of the systolic phase. It is proposed by the authors that the development of a syrinx is related to increased pressure on the spinal cord. If we compare the studies of Heiss [21] and Pinna [28], the systolic phase would be longer. This would result in the increased duration of barometric stress on the medullary cord. On the contrary, in patients without a syrinx, the duration of barometric stress on the medullary cord is not sufficient to generate the development of a syrinx.

Our study shows a more important hydrodynamic and craniospinal hemodynamic alteration in CS associated with a syrinx. These alterations may be secondary to the arteriovenous delay, which would be lengthened and thus act on the temporal distribution between systolic and diastolic durations. If there is an arteriovenous alteration related to an alteration to venous vascular compliance, the CSF should play a role as a compensatory mechanism. In CS, the CSF has, by definition, a disturbance of free flow. This can lead to the formation of a syrinx. The genesis of this syrinx would be potentially secondary to an alteration to CSF flow but also to an alteration to venous blood flow.

In many CSF pathologies, potential alterations in venous drainage have been demonstrated [11,31,32,33,34]. Alterations in venous drainage have not been explored in CS. This new approach may guide us towards a new pathophysiological understanding.

### 4.3. Limits and Perspectives

Our groups were heterogeneous in size. This is due to the recruitment methods, which were retrospective. This is a rare orphan disease. The presence of a syrinx is also rare. This study shows the interest of a prospective analysis of this major aspect of this still unexplored pathology.

In addition to a prospective analysis, we propose a temporal analysis of hemodynamics and hydrodynamics. We have observed profiles that may be linked to an increase in cardiac systole duration. We propose an analysis of arteriovenous delay in populations with and without a syrinx.

## 5. Conclusions

We have shown that there are differences in hemodynamic and hydrodynamic interactions depending on the presence or absence of an associated syrinx in patients with CS. PCMRI allows for the quantification of variations in intracranial vascular volume and the response of the CSF. CSF is a passive liquid in terms of its flow and responds to physical laws such as pressure gradients and resistance to flow, thus also responding to the flow surface. Disturbances related to venous drainage can have several origins and can be both an element of the genesis of syrinxes in CS and a consequence. These disturbances of hemohydrodynamic interactions could open a new field of pathophysiological but also clinical exploration.

## Figures and Tables

**Table 1:** Acquisition parameters of phase-contrast MRI (PCMRI).

	PCMRI
FOV (cm^2^)	14 × 14 or 16 × 16
Resolution (mm^2^)	0.6 × 0.6
Thickness (mm^2^)	2
Flip angle (degree)	30
SENSE	1.5
TE (ms)	Minimum
TR (ms)	Minimum
Number of images	32
Acquisition time (s)	50–115
Number of images per cycle	32

**Table 2:** Population characteristics. M: male; F: female; s: syrinx; wo.s: without syrinx.

Patients Parameters	
Sex ratio (M/F)	2/26
Syrinx ratio (s/wo.s)	9/19
Age (years)	37 ± 12

**Table 3:** Hemodynamics and hydrodynamics in Chiari with or without a syrinx. CC: cardiac cycle. SV_CSF-aqu_ corresponds to the stroke volume of CSF oscillating through the aqueduct during one cardiac cycle. SV_CSF-FM_ corresponds to the stroke volume of CSF oscillating through the foramen magnum during one cardiac cycle. SV_tonsils_ corresponds to the stroke volume of the tonsils that oscillates through the foramen magnum during one cardiac cycle. This is due to the pulsatility of the tonsils. SV_CSF-PPC_ corresponds to the stroke volume of CSF oscillating through the prepontine cistern (PPC) during one cardiac cycle. SV_CSF-cerv_ corresponds to the stroke volume of CSF oscillating through the upper cervical SAS at the level of the C2C3 intervertebral disc during one cardiac cycle. SV_syrinx_ corresponds to the stroke volume of CSF oscillating into the syrinx during one cardiac cycle. CBF represents mean cerebral blood flow during one cardiac cycle. SV_blood_ corresponds to the volume of blood which oscillates into the cranial compartment during one cardiac cycle. α factor represents the venous correction factor, which corresponds to the venous drainage repartition between jugular veins and other peripheral veins. Statistical test used: Student’s *t*-test.

Flow Parameters	CS with a Syrinx	CS without a Syrinx	*p* Value
SV_CSF-aqu_ (µL/CC)	47 ± 35	39 ± 26	0.55
SV_CSF-FM_ (µL/CC)	484 ± 193	464 ± 208	0.50
SV_tonsils_ (µL/CC)	181 ± 106	181 ± 128	0.99
SV_CSF-PPC_ (µL/CC)	365 ± 169	309 ± 212	0.55
SV_CSF-cerv_ (µL/CC)	587 ± 167	584 ± 162	0.96
SV_syrinx_ (µL/CC)	47 ± 7	/	/
CBF (mL/min)	728 ± 192	685 ± 133	0.52
SV_blood_ (mL/CC)	0.83 ± 0.19	0.81 ± 0.27	0.79
α-factor	1.46 ± 0.26	1.49 ± 0.17	0.72

**Table 4:** Correlation between cerebral vascular stroke volume and hydrodynamic parameters. Study of the correlations between hemodynamic and hydrodynamic parameters was conducted in the type I Chiari malformation (CS) with a syrinx group (CS/syrinx group), the CS without a syrinx group (CS group) and the overall population (CS + CS/syrinx groups). SV_CSF-FM_ corresponds to the stroke volume of CSF oscillating through the foramen magnum during one cardiac cycle. SV_tonsils_ corresponds to the stroke volume of the tonsils that oscillates through the foramen magnum during one cardiac cycle. SV_CSF-PPC_ corresponds to the stroke volume of CSF oscillating through the prepontine cistern (PPC) during one cardiac cycle. SV_CSF-cerv_ corresponds to the stroke volume of CSF oscillating through the upper cervical SAS at the level of the C2C3 intervertebral disc during one cardiac cycle. SV_blood_ corresponds to the volume of blood oscillating into the cranial compartment during one cardiac cycle. Statistical test: Pearson correlation.

	CS with a Syrinx R^2^ (*p*-Value)	CS without a Syrinx R^2^ (*p*-Value)	Overall CS R^2^ (*p*-Value)
SV_blood_ vs. SV_CSF-FM_	0.32 (0.39)	0.38 (0.10)	0.36 (0.15)
SV_blood_ vs. SV_CSF-PPC_	0.48 (0.18)	0.15 (0.51)	0.23 (0.22)
SV_blood_ vs. SV_tonsils_	0.17 (0.64)	−0.22 (0.34)	−0.14 (0.47)
SV_blood_ vs. SV_CSF-cerv_	0.04 (0.89)	0.67 (<0.01)	0.50 (<0.01)

**Table 5:** Correlation between the α venous correction factor and hydrodynamic parameters. Study of the correlations between venous drainage and hydrodynamic parameters was conducted in the Chiari syndrome (CS) with a syrinx group, the CS without a syrinx group and the overall population. SV_CSF-FM_ corresponds to the stroke volume of CSF oscillating through the foramen magnum during one cardiac cycle. SV_tonsils_ corresponds to the stroke volume of the tonsils that oscillates through the foramen magnum during one cardiac cycle. This is due to the pulsatility of the tonsils. SV_CSF-PPC_ corresponds to the stroke volume of CSF oscillating through the prepontine cistern (PPC) during one cardiac cycle. SV_CSF-cerv_ corresponds to the stroke volume of CSF oscillating through the upper cervical SAS at the level of the C2C3 intervertebral disc during one cardiac cycle. α factor corresponds to the venous drainage repartition. CBF_a_ correspond to the mean arterial blood flow.

	CS with a Syrinx	CS without a Syrinx	Overall CS
α factor vs. CBF_a_	0.46 (0.21)	0.36 (0.13)	0.40 (0.03)
α factor vs. SV_CSF-FM_	−0.61 (0.04)	−0.23 (0.32)	−0.37 (0.04)
α factor vs. SV_tonsils_	0.47 (0.20)	0.30 (0.20)	0.34 (0.06)
α factor vs. SV_CSF-PPC_	−0.40 (0.28)	−0.31 (0.24)	−0.17 (0.37)
α factor vs. SV_CSF-cerv_	−0.80 (<0.01)	−0.05 (0.81)	−0.37 (0.05)

## Data Availability

Not applicable.
